# Spontaneous, Voluntary, and Affective Behaviours in Rat Models of Pathological Pain

**DOI:** 10.3389/fpain.2021.672711

**Published:** 2021-07-01

**Authors:** Peter Draxler, Aurora Moen, Karolina Galek, Ani Boghos, Dariga Ramazanova, Jürgen Sandkühler

**Affiliations:** ^1^Division of Neurophysiology, Center for Brain Research, Medical University of Vienna, Vienna, Austria; ^2^Center for Medical Statistics, Informatics and Intelligent Systems (CeMSIIS) Section for Medical Statistics, Medical University of Vienna, Vienna, Austria

**Keywords:** non-evoked pain, rodent behaviour, affective behaviour, pain models, behaviour test

## Abstract

In pain patients affective and motivational reactions as well as impairment of daily life activities dominate the clinical picture. In contrast, many rodent pain models have been established on the basis of mechanical hypersensitivity testing. Up to today most rodent studies on pain still rely on reflexive withdrawal responses only. This discrepancy has likely contributed to the low predictive power of preclinical pain models for novel therapies. Here, we used a behavioural test array for rats to behaviourally evaluate five aetiologically distinct pain models consisting of inflammatory-, postsurgical-, cephalic-, neuropathic- and chemotherapy-induced pain. We assessed paralleling clinical expressions and comorbidities of chronic pain with an array of behavioural tests to assess anxiety, social interaction, distress, depression, and voluntary/spontaneous behaviours. Pharmacological treatment of the distinct pain conditions was performed with pathology-specific and clinically efficacious analgesics as gabapentin, sumatriptan, naproxen, and codeine. We found that rats differed in their manifestation of symptoms depending on the pain model and that pathology-specific analgesics also reduced the associated behavioural parameters. Based on all behavioural test performed, we screened for tests that can discriminate experimental groups on the basis of reflexive as well as non-sensory, affective parameters. Together, we propose a set of non-evoked behaviours with a comparable predictive power to mechanical threshold testing for each pain model.

## Introduction

Chronic pain affects up to 30% of the western adult population and shows a rising incidence over the past decades (1–3). Translational failures from promising preclinical studies to successful clinical trials has led to a remarkable lack of novel analgesics (4–6). A factor likely contributing to this situation is the fundamental difference between preclinical and clinical assessment of chronic pain conditions.

Clinical trials rely on self-reported outcome measures such as ongoing/persistent pain intensity, quality of life, and everyday functioning (7–9). This assessment also includes frequently observed psychological comorbidities of chronic pain. Consequently, the evaluation of analgesic efficacy in pain patients is based on the spectrum of these parameters (10–12). In preclinical rodent models, however, threshold tests for mechanical- and thermal stimuli are still widely used as the sole affirmative markers for pathological pain states and as indicators for analgesic efficacy (13–15). An overemphasis on reflexive responses might neglect paralleling affective behavioural components of chronic pain observed in humans, and could therefore represent a core limitation of clinical translation (16). Recent developments in basic research aim to meet clinical pain assessment by the evaluation of additional behavioural parameters. Among these, facial expressions as an indicator for ongoing pain, and tunnel-burrowing as marker for the overall functioning and ability to work, have been proposed (17, 18). While these tests clearly advance our understanding of rodent pain behaviour, they still reduce, like threshold testing and unlike clinical evaluation, a complex pathological state to a single behavioural parameter. In other research areas of neurological diseases, rodent testing batteries, based on well-established behavioural paradigms with high face-validity, have been introduced as tools to improve translational success (19, 20).

Here, we behaviourally evaluated five aetiologically distinct rat models of inflammatory-, postsurgical-, cephalgia-, neuropathic-, and chemotherapy-induced pain for their manifestations of comorbidities common in patients with chronic pain (21–24). We implemented a behavioural testing sequence assessing both sensory gain (classical reflexive threshold tests) and affective components of pain, including social interaction (three chamber apparatus), anxiety (elevated plus maze), depression (splash test, saccharine preference), distress (nest building), and voluntary/spontaneous behaviour (automated classification). We further screened for model specific, non-evoked, behavioural tests that could assess the treatment efficacy of analgesics on a preclinical level with a similar accuracy as reflexive measurements. To broaden the observational view on analgesic effects, we chose three classes of compounds for the treatment of the induced pain conditions: (1) Preclinical and clinical successful analgesics with well-established numbers needed to treat (NNT) (naproxen, sumatriptan, codeine and gabapentin). (2) A compound belonging to a substance-class with preclinical promising results but with low clinical success (aprepitant, a neurokinin-1-receptor antagonist). (3) A compound which has not yet been tested in a clinical setting for the particular pathology (phenytoin). We chose phenytoin as a treatment compound, as its pharmacological profile suggests that it might counteract the oxaliplatin-induced pathology (25, 26). We show that although aetiologically distinct pain models share the development of mechanical hypersensitivity, they differ in their behavioural symptom manifestations. We further describe, in each pain model, a set of non-evoked behavioural parameters that could aid hypersensitivity testing in the assessment of pain behaviour and treatment efficacy of analgesics in rodents.

## Materials and Methods

### Animals

We used male Sprague-Dawley rats weighing 150–200 grammes (age: 31–38 days) at the start of acclimatisation. Animals were group-housed (3 individuals) under standard conditions with *ad libitum* access to food (standard rat chow pellets) and water under a standard 12-h light-dark regime.

### Behaviour

#### Testing Conditions

On two consecutive days before testing, and on each testing day, rats were habituated for at least 1 h to the testing room. The testing- and housing-room conditions, unless stated otherwise, were as follows: room temperature 21 ± 1°C, humidity 40–60%, luminance ~500 lx. Experiments were performed during the light cycle. Animals were tested by male- and female experimenters.

#### Carrageenan-Induced Inflammatory Pain

Rats were anaesthetised with 1.25–1.5% isoflurane, and 100 μl 1% λ-carrageenan (Sigma) dissolved in saline was injected unilaterally into the plantar hind-paw to model inflammatory pain. The vehicle control group received saline injections (27).

#### Incision Model of Postsurgical Pain

Incision of a hind paw, a model of postoperative pain, was performed under 1.25–1.5% isoflurane anaesthesia, as previously described (21). A unilateral 1-cm longitudinal incision was made with a number 10 scalpel blade through the skin and fascia of the plantar hind-paw. The plantaris muscle was elevated with a forceps and incised longitudinally. The skin was opposed with three prolene 4/0 (ethicon) and the wound site covered with neomycin. The control-group procedure consisted of anaesthesia, sterile preparation, and neomycin application on the hind paw.

#### Nitroglycerine-Induced Cephalgia

Rats received a single *i.p*. injection (10 mg/kg) of a nitroglycerine solution (Nitro POHL, Pohl Boskamp) to model cephalgia (22). Animals of the control group received vehicle injections.

#### Chronic Constriction Injury

Briefly, rats were deeply anaesthetised with 1.25–1.5% isoflurane and the right sciatic nerve was exposed at the mid-thigh level. After removal of adherent tissue, three loose ligatures with chromic gut 6/0 (SMI) were tied 1 mm apart around the nerve, proximal to the trifurcation (28). Muscle and skin were closed with sutures (Silkam 6/0 and prolene 4/0, respectively; Ethicon) and wound clips. In control-animals, all steps were identical, except that the sciatic nerve was exposed but not ligated.

#### Oxaliplatin-Induced Neuropathy

Oxaliplatin was used as a model for chemotherapy-induced neuropathy (23). Rats received two treatment cycles, each consisting of 5 consecutive daily *intra peritoneal* (*i.p*) injections of 3 mg/kg oxaliplatin (Tocris; cumulative dose: 30 mg/kg) with 5 days of rest in between both treatment cycles. For injections, oxaliplatin was dissolved in a 5% glucose solution (Braun) at a concentration of 0.5 mg/ml. Vehicle-treated control animals received glucose injections. The weight of the animals was monitored daily during the testing period.

#### Administration of Therapeutics

**Figure 1:**
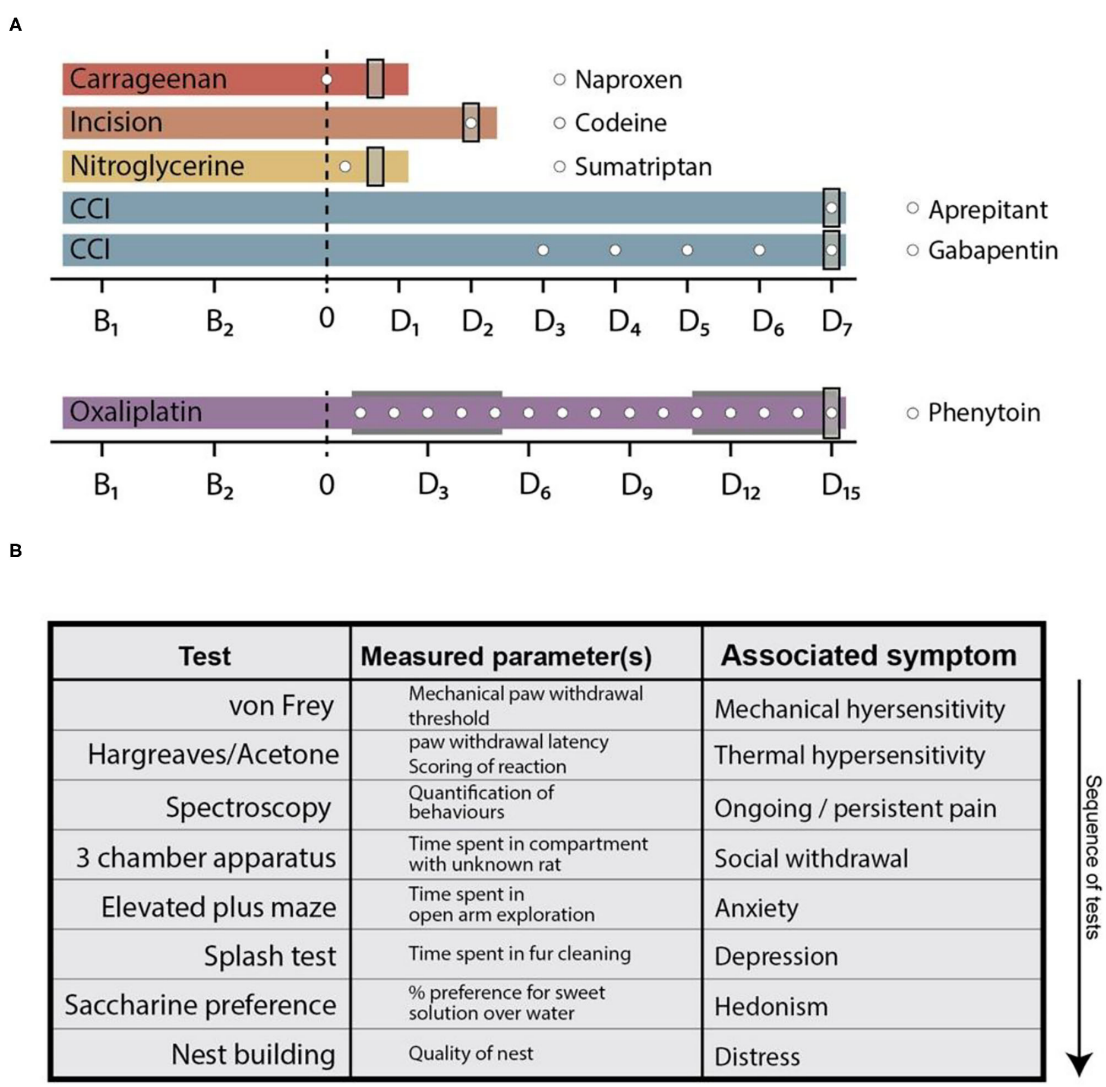
Behavioural time courses of the used pain models and testing array. **(A)** Behavioural time courses of the pain models used in this study. Dashed line at time point 0 indicates pain model induction. In the oxaliplatin model, the two grey areas indicate cyclic injections of oxaliplatin. Time points on the x-axis (B and D) indicate threshold testing days for the von Frey- and plantar heat test (the planar heat test was substituted by the acetone test in the oxaliplatin pain model). “B_x_”—legends indicate baseline testing days, whereas “D_x_”—legends indicate testing days after pain model induction. White circles depict time points of analgesic administration. The used compounds are shown in the legend next to the corresponding pain models. Grey rectangles at the end of each time course indicate the time point of the complete behavioural array. **(B)** Tabular illustration of the tests in the behavioural array. The list represents the sequence in which the tests were performed and indicated *via* the arrow. CCI, chronic constriction injury.

Naproxen (Tocris, 10 mg/kg), codeine (St. Martins Pharmacy, 7 or 30 mg/kg), sumatriptan (Tocris, 10 mg/kg), and gabapentin (Sigma, 30 mg/kg) were dissolved in physiological saline (Braun) and administered *via* a single *i.p*. injection. Phenytoin (Sigma, 15 mg/kg) and aprepitant (MedChemExpress, 20 mg/kg) were dissolved in PEG-400 (Sigma) and delivered *via i.p*. injections. Drug injection schedules are detailed in Figure 1A: naproxen, sumatriptan, and aprepitant were administered once on the last testing day. Codeine was administered at the last testing day twice (1 h before testing and when testing was concluded–before the night cycle). Phenytoin was administered once daily over a 15 day period, starting with the first injection of oxaliplatin. Gabapentin was injected once per day over a period of 5 consecutive days starting 2 days after CCI-surgery.

#### von Frey Test

Rats were placed in acrylic glass enclosures on a wire mesh floor and allowed to habituate for at least 30 min prior to testing. Mechanical sensitivity was tested by applying calibrated von Frey filaments (Stoelting) to the plantar surface of the hind paw. The “up and down paradigm” established by Chaplan et al. (14) was used, starting with the 2 g filament and 6 filament applications.

#### Plantar Heat Test

Animals were placed in acrylic glass enclosures on the base of the plantar heat apparatus (Stoelting) and allowed to habituate to the test environment for at least 30 min before testing. Heat sensitivity was measured by application of radiant heat (100–130 mW/cm^2^) to the plantar surface of the hind-paw. To avoid tissue damage, the cutoff latency was set to 20 s. Measurements were taken alternatingly on both hind paws with at least 5 min as inter-stimulus interval. For each testing time point both hind paws were stimulated three times (13).

#### Acetone Test

Rats were placed in acrylic glass enclosures on an elevated wire mesh and allowed to habituate to the environment for 30 min before testing. Using a micropipette, 100 μl of acetone were applied to the plantar hind paw of the animal. The first 10 s of the response were disregarded due to the mechanical co-stimulation of the paw. The behaviour of the animals during the following 60 s was observed and scored (29): 0, no reaction; 0.5, glancing at the paw; 1, withdrawal or paw lift; 1.5, scratching and/or paw licking and/or paw bending; 2, brisk paw withdrawal; 3, scratching over an extended time period; 4, flicking; 5, extended licking of the stimulated paw.

#### Behavioural Spectroscopy

Rats were tested individually for a period of 10 min in the behavioural spectroscopy apparatus (Behavioural Instruments) without prior exposure to this setup (30). The enclosure size was 40 × 40 × 45 cm and light intensity in the apparatus was ~10 lx. Animal behaviour was videotaped and analysed in real-time *via* Viewer3 and the spectroscopy plug-in program (Biobserv). A customised classification software was used for the detection of rat behaviour (Supplementary Figure 1). Based on the video-, infrared-, and vibration-signals, the following behaviours were detected by the algorithm: still (motionless without head movements), walk (slow movement with flat posture), trot (medium movement with arched posture), run (fast movement with arched posture), time spent in locomotion, rearing (supported and unsupported standing on the hind paws), orientation (head movements/extension associated with olfactory and visual orientation), grooming (grooming behaviour on head, face, flanks, abdomen, and back), limb-directed behaviour (licking and scratching of the paws), general tracklength (cm), tracklength in the central field (cm, virtual central 20 × 20 cm square), activity (percent of time in locomotion), and average locomotion velocity (cm/s).

#### 3 Chamber Social Interaction Apparatus

A custom build 3 chamber apparatus was used (30 × 80 cm for each of the 3 compartments; IST, Austria) (31). A stranger animal of the same age, which has been habituated to the testing setup on two consecutive days (30 min each), was present in one of the enclosure-cages (20 cm in diameter). The corresponding enclosure cage in the non-neutral compartment was left empty. The enclosure of the stranger animal was alternated after each test session. Test animals were allowed to habituate in the neutral chamber for 5 min. After the opening of both doors to the stranger- and the empty-compartment, test animals were allowed to explore the full setup for 10 min. Total time spent in each compartment was tracked *via* a USB camera (Stoelting) and the Viewer3 software (Biobserv). Test animals have not been habituated to the setup prior to testing. Room light intensity was set to ~200 lx.

#### Elevated Plus Maze

A custom made elevated plus maze was used to test anxiety (50 cm height, 10 × 50 cm per arm; IST, Austria). Animals were, without prior habituation, placed on the middle crossing of the maze and allowed to explore the setup for 10 min (32). The behaviour of the animals was video-recorded and evaluated *via* a rodent tracking software (Viewer3, Biobserv). The light intensity was set to 200 lx.

#### Splash-Test

Rats were sprayed with a 10% sucrose (Sigma-Aldrich) solution on their back coat, and were subsequently transferred into the spectroscopy apparatus. The grooming behaviour of single animals was videotaped and automatically categorised and quantified in the spectroscopy apparatus over an observation period of 5 min (33).

#### Saccharin Preference Test

Prior to testing, the water bottle of the animal's home cage was replaced by a 0.25% saccharine solution (Sigma-Aldrich) for 2 h on two consecutive days. At the testing time point, rats were single housed and provided with two liquid bottles, one filled with water and one filled with a 0.1% saccharin solution. After 12 h, the position of the bottles was swapped. After another 12 h (a total of 24 h) the water and saccharine consumption was measured (34). The saccharine preference (percent) was calculated as follows: 100 × (consumed saccharine, ml)/[(consumed saccharine, ml) + (consumed water, ml)].

#### Nest-Building

Animals were single housed over a period of 24 h and provided with 6 Nestlet™ cotton pads each (Ancare) (35). The nest quality was scored as follows: 0, nesting material not manipulated, possibly dragged around the cage; 1, nesting material slightly manipulated, most of nesting material intact, possibly few shreds picked out; 2, nesting material noticeably manipulated, <80% of nesting material intact, shreds spread around or in one area; 3, noticeable nest site, <80% of nesting material intact, shreds placed mostly in nest site; 4, nesting material not intact, shreds encasing the nest <50%; 5, nesting material not intact, cotton-shreds encasing the nest.

#### Study Design

The behavioural time courses of the pain models and the application regimens of the analgesics are depicted in Figure 1. Briefly, all models consisted of two baseline testing days in which the mechanical- and heat-withdrawal thresholds were assessed (von Frey and plantar heat test; plantar heat was substituted with the acetone test in the oxaliplatin model of chemotherapy-induced neuropathy). Following the model induction, a cohort of animals in pain models were subjected to analgesic administration and at least once additionally tested for their mechanical- and thermal-responses (carrageenan- and nitroglycerine-model). Rats subjected to CCI surgery or cyclic oxaliplatin injections were followed over a longer time period with mechanical- and thermal-sensory tests (CCI: 7 days, daily testing; incision: 2 days, daily testing; oxaliplatin: 15 days, testing at 3 day intervals). At the last testing day of each time course, all animals were subjected to the behavioural array where each animal was tested on all behaviour assays in the same test sequence (Figure 1B). The time-points where chosen based on established time-courses of mechanical hypersensitivities: carrageenan-induced inflammatory pain, 3 h (24); incision model of postsurgical pain, 2 days (21), nitroglycerine-induced-cephalgia, 3 h (22); chronic constriction injury, 7 days (28); oxaliplatin model of chemotherapy-induced neuropathy, 15 days (23). Signs of non-well-being as unclean fur, loss of body weight, abnormal faecal consistency, injuries or infections were specified as exclusion criteria. From a total of 198 rats, one animal was excluded from the study. Rats were randomly allocated to the treatment groups before initial testing. Experimenters were blinded to the pain model and treatment regimen of the rats until data collection was completed.

### Statistical Analysis

All data are expressed as mean ± SEM unless stated otherwise. All statistical analyses were conducted using GraphPad Prism 6. A repeated measures (RM) two-way ANOVA with Bonferroni's *post-hoc* test was used to compare treatment effects in the behaviour time course tests (von Frey, plantar heat) and a non-parametric analysis of longitudinal data was used to compare treatment effects in the acetone time-course experiments (36). Single time point group-differences in affective behavioural tests were analysed *via* one-way ANOVA with Bonferroni's *post-hoc* correction or a Kruskal-Wallis test with Dunn's multiple comparison test was used (vehicle vs. respective treatment groups). In case of unequal group sizes, Dunnett's *post-hoc* correction was performed. To assess accuracy of parameters to discriminate between control- and pain-groups, firth logistical regression was performed (37). In case of perfect separation, Cutoff was defined based on the logistic regression predictions as the mean value of the smallest prediction in the pain group and the largest prediction in the control group. In case of no perfect separation, the Cutoff is obtained by maximising the sum of specificity and sensitivity. Using the calculated Firth logistic regression models and the corresponding Cutoff, the values of the treatment group (or groups) have been classified either as control or pain group. All statistical analyses reported here were done using R (using package logistf for Firth regression and nparLD for longitudinal non-parametric analysis).

### Study Approval

All procedures were conducted in accordance with guidelines set by the Ethical Committees for the use of laboratory animals at the Medical University of Vienna and the Austrian Ministry for Science and Research (BMWF). These experiments conform to the standards as specified by the European Union (EU) and the International Association for the Study of Pain (IASP).

## Results

### Naproxen Reduces Most Behavioural Manifestations of Carrageenan-Induced Inflammatory Pain

**Figure 2:**
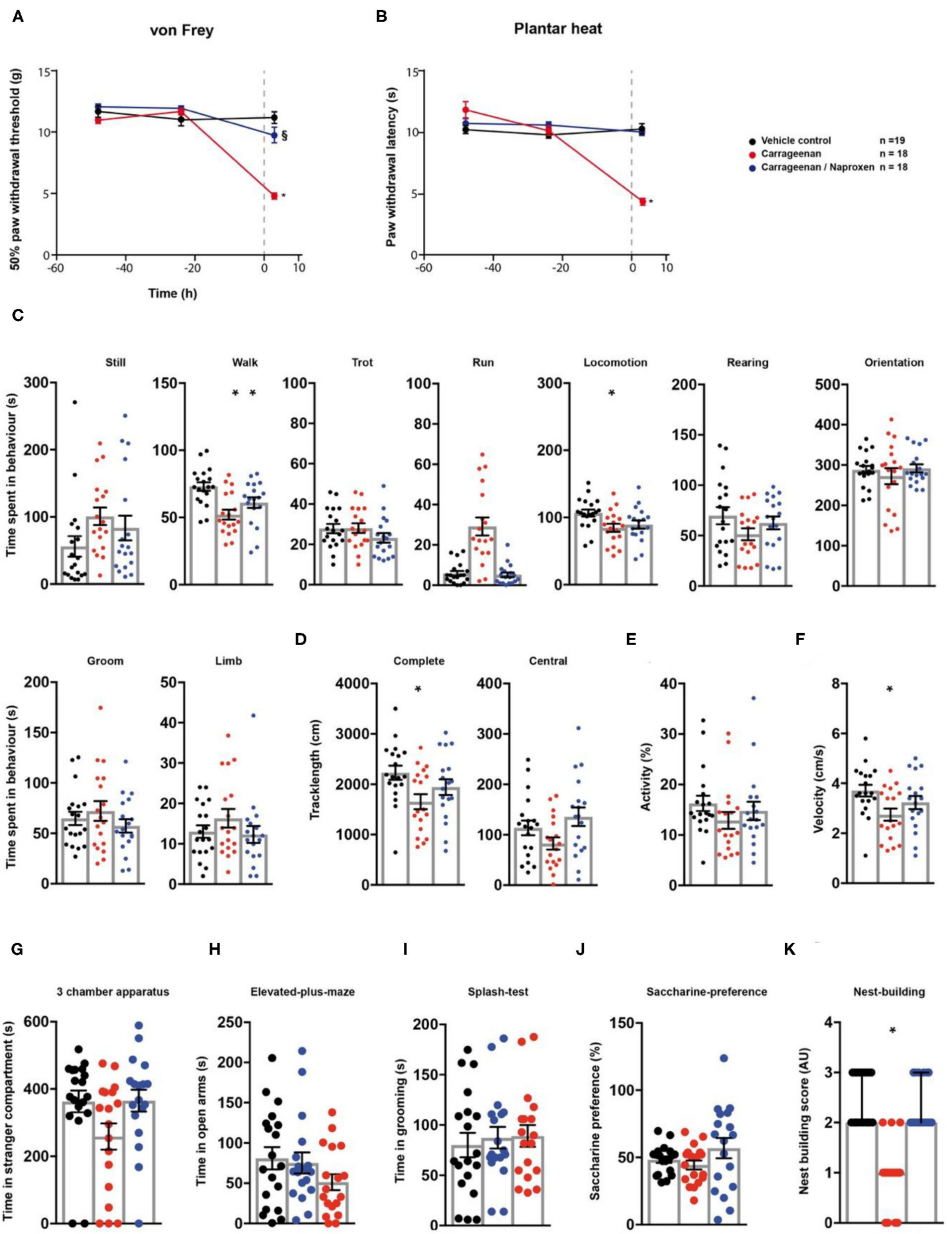
Naproxen significantly reduces pain-related behavioural parameters in a model of carrageenan-induced peripheral inflammation. Carrageenan- (red traces/columns), vehicle- (black traces/columns), and carrageenan/naproxen-treated rats (blue traces/columns) were tested on two baseline- and one time point post injection for their responses to mechanical- and heat stimuli. Carrageenan and naproxen injections were performed at time point 0, as indicated by the grey dashed line **(A,B)**. At the testing time point post injection (3 h), all groups were subjected to the testing sequence **(C–K)**. **(A)** von Frey test/mechanical paw withdrawal thresholds. **(B)** Plantar heat test/radiant-heat induced paw-withdrawal latency. **(C–F)** Automated classification of voluntary behaviours in the spectroscopy apparatus. The parameters are ordered in general behaviours **(C)**, tracklength **(D)**, overall activity **(E)**, and velocity **(F)**. **(G)** 3 chamber apparatus/time spent in a compartment with an unknown animal. **(H)** Elevated plus maze/time spent in open arm exploration. **(I)** Splash-test/time spent in induced grooming behaviour. **(J)** Saccharine preference. **(K)** Nest building scoring (median ± 95% confidence interval, Kruskal-Wallis test with Dunn's multiple comparison test). Data are expressed as mean ± SEM and analysed using a RM two-way ANOVA **(A,B)**, followed by Bonferroni's correction. For single time point behavioural tests, a one-way ANOVA with Bonferroni's correction was used (**C–K**, *n* = 19 for vehicle control, *n* = 18 for the carrageenan- and the carrageenan/naproxen treated group). * indicates a significant difference to the vehicle control (*p* < 0.05); § indicates a significant difference to the carrageenan-group (*p* < 0.05). For detailed *p*-values of group comparisons, see Table 1.

First, we evaluated the effect of naproxen on carrageenan-induced inflammatory pain *via* the behavioural testing sequence (Figures 1A,B). After two baseline testing days for the animals' threshold responses to mechanical- and thermal stimuli assessed *via* the von Frey- and the plantar heat test, rats received an *intra-plantar (i.pl.)* injection of carrageenan to model peripheral inflammation (24). A cohort of animals subjected to carrageenan-induced inflammatory pain, received in addition a single *i.p*. injection of naproxen (10 mg/kg). Rats in inflammatory pain showed significantly reduced mechanical paw withdrawal thresholds in the von Frey test, 3 h post administration (Figure 2A; *p* < 0.0001; Table 1). Lowering in mechanical paw withdrawal thresholds was alleviated by naproxen (Figure 2A; *p* < 0.0001; Table 1). Similarly, heat hypersensitivity developed in carrageenan-injected rats, which was reversed by naproxen co-injection (Figure 2B; both: *p* < 0.0001; Table 1). Next, all three treatment groups were tested in the spectroscopy apparatus. A customised classification software was used for the detection of behaviour (Supplementary Figure 1). Carrageenan-treated animals depicted an overall altered locomotive pattern, which included a significant decrease in the time spent in walking (*p* = 0.0003) and in general locomotion (*p* = 0.017; Figure 2C; Table 1), when compared to the vehicle control. Further, the total track length (*p* = 0.016) and the locomotion velocity (*p* = 0.015) were significantly reduced in animals with carrageenan-induced peripheral inflammation (Figures 2D,F; Table 1). The carrageenan-mediated effects were reduced by naproxen injections for all parameters (*p* > 0.05), except for the time spent in walking (*p* = 0.045; Figures 2C–F; Table 1).

**Table 1:**
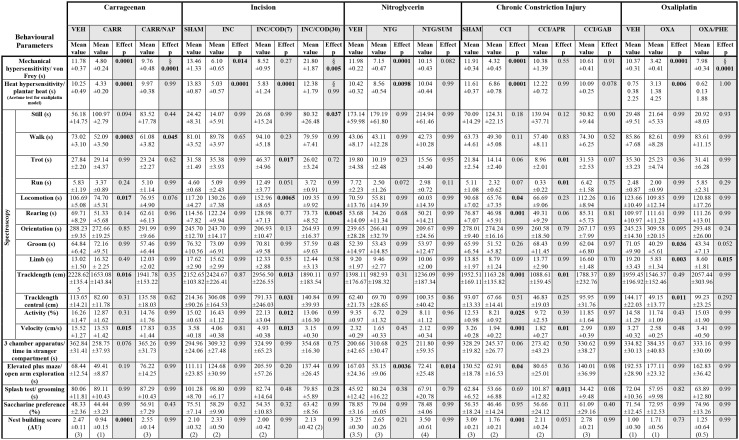
Response values and statistics of pain models ant treatment regimens.

*Table illustrates mean response values ± SEM obtained in the different behavioural paradigms, as indicated, as well as p-values obtained in statistical tests. Data were analysed using a RM two-way-ANOVA with time and group as dependent variables with Bonferroni's correction (von Frey and plantar heat). All other comparisons were performed with a one-way ANOVA with Bonferroni's correction. For the nest building parameter a Kruskal-Wallis test with Dunn's multiple comparison test was used. Median values are indicated in brackets. ^§^indicates a significant difference to the pain model. Bold values indicate statistical significance*.

Next, animals were tested for affective components of inflammatory pain. Short-term peripheral inflammation induced by carrageenan- and carrageenan/naproxen administration had no detectable effect on the social behaviour, open arm exploration in the elevated plus maze, grooming in the splash test, or saccharine preference (Figures 2G–J; Table 1; *p* > 0.05 for all tests and treatment groups). However, rats in carrageenan-induced inflammatory pain showed a significant decrease of nest quality in the nest building task (*p* = 0.0001; Figure 2K; Table 1). This deficit was absent in animals which received carrageenan/naproxen co-administration (*p* > 0.99; Figure 2A; Table 1). Together, these results show a behavioural impact of carrageenan-induced inflammatory pain on non-evoked behavioural parameters and distress beyond mechanical- and thermal hypersensitivities. The behavioural test battery captured the clinically well-established potency of naproxen as an analgesic for inflammatory pain.

### Codeine Has a Dose Dependent Effect on Post-surgical Pain-Induced Hypersensitivities

**Figure 3:**
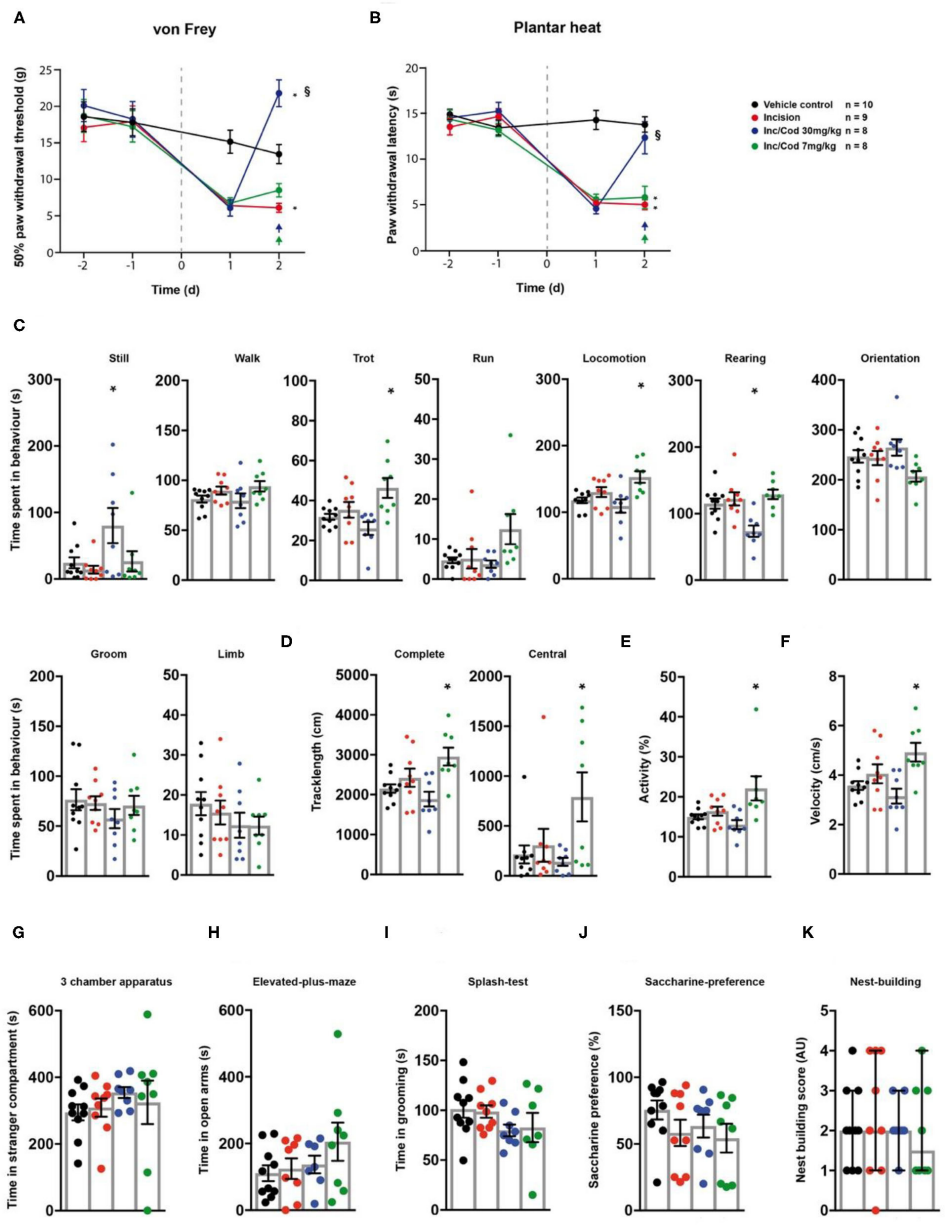
Postsurgical incision model shows low levels of behavioural modulation. Incision- (red traces/columns), vehicle- (black traces/columns), incision/codeine 7 mg/kg (green traces/columns), and incision/codeine 30 mg/kg—treated (blue traces/columns) rats were tested on two baseline- and two time points post injection for their responses to mechanical- and heat stimuli. Incision surgery was performed at time point 0, as indicated by the grey dashed line **(A,B)**. Codeine treatment groups received two bolus injections on the last testing day (evening/morning; 7 mg/kg each). At this time point, all groups were subjected to the testing sequence **(C–K)**. **(A)** von Frey test/mechanical paw withdrawal thresholds. **(B)** Plantar heat test/radiant-heat induced paw-withdrawal latency. **(C–F)** Automated classification of voluntary behaviours in the spectroscopy apparatus. The parameters are ordered in general behaviours **(C)**, tracklength **(D)**, overall activity **(E)**, and velocity **(F)**. **(G)** 3 chamber apparatus/time spent in a compartment with an unknown animal. **(H)** Elevated plus maze/time spent in open arm exploration. **(I)** Splash-test/time in induced grooming behaviour. **(J)** Saccharine preference. **(K)** Nest building scoring (median ± 95% confidence interval, Kruskal-Wallis test with Dunn's multiple comparison test). Data are expressed as mean ± SEM and analysed using a RM two-way ANOVA **(A,B)**, followed by Bonferroni's correction. For single time point behavioural tests, a one-way ANOVA with Bonferroni's correction was used (**C–K**, *n* = 10 for the control group, *n* = 9 for the incision model, *n* = 8 for the incision/codeine 7 mg/kg—and the incision/codeine 30 mg/kg groups). * indicates a significant difference to the vehicle control (*p* < 0.05); § indicates a significant difference to the incision-group (*p* < 0.05). For detailed *p*-values of group comparisons, see Table 1.

Next, we evaluated the effect of codeine (*i.p*. injection at 7 or 30 mg/kg) on incision-induced acute postsurgical pain *via* the testing sequence (Figures 1A,B). A unilateral incision at the plantar surface of the hind-paw was performed to model postsurgical pain (21). Rats in postsurgical pain showed significantly lowered mechanical paw withdrawal thresholds in the von Frey test, 2 days post model induction (*p* = 0.014; Figure 3A; Table 1). These reductions were mitigated in rats which received codeine at 30 mg/kg (*p* = 0.005), but not at 7 mg/kg (*p* = 0.27; Figure 3A; Table 1). Similarly, a significant heat hypersensitivity developed in rats with a paw-incision when compared to the control -group (*p* = 0.0001), which was reduced by codeine treatment at 30 mg/kg (*p* = 0.99), but not with 7 mg/kg (*p* = 0.0001; Figure 3B; Table 1). These effects of codeine at 30 mg/kg compared to 7 mg/kg on the thresholds tests may, however, be related to side-effects on motor behaviour as the administration of codeine at 30 mg/kg also significantly increased still behaviour (*p* = 0.037) and significantly reduced rearing behaviour (*p* = 0.0045) in the spectroscopy tests (Figure 3C; Table 1). In contrast, codeine at a dose of 7 mg/kg significantly increased locomotion parameters as trotting, general locomotion, track length in the complete and central field, activity and velocity (Figures 3C–F; *p* < 0.5, Table 1). Neither the incision model of acute postsurgical pain nor the administration of codeine had any effect on the remaining of the behavioural tests (*p* > 0.05; Figure 3; Table 1).

### Behavioural Manifestations of Nitroglycerine-Induced Cephalgia

**Figure 4:**
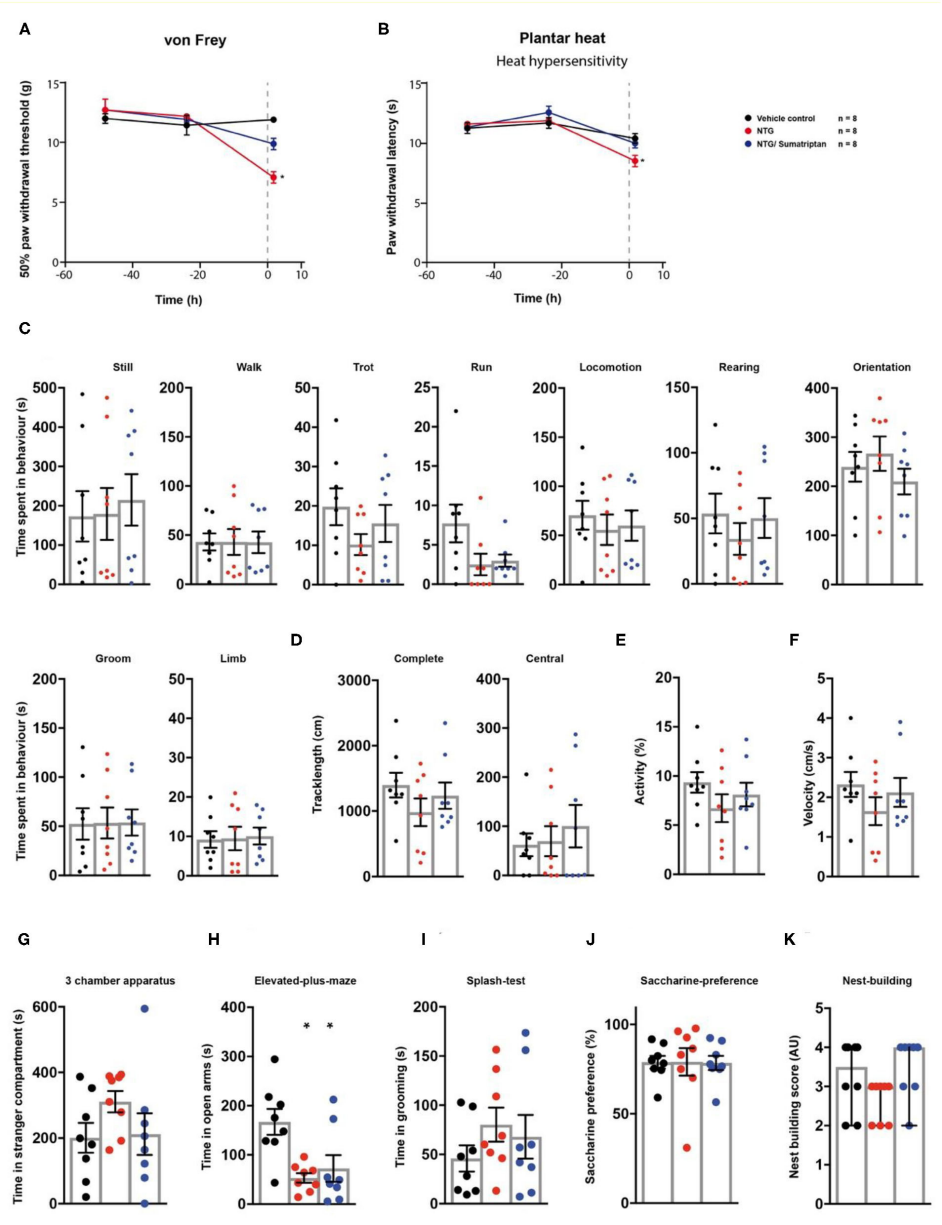
Sumatriptan reduces nitroglycerine-induced hypersensitivities. Nitroglycerine- (red traces/columns), vehicle- (black traces/columns), and nitroglycerine/sumatriptan-treated (blue traces/columns) rats were tested on two baseline- and one time point post injection for their responses to mechanical- and heat stimuli. Nitroglycerine and sumatriptan injections were performed at time point 0, as indicated by the grey dashed line **(A,B)**. At the testing time point post injection, all groups were subjected to the testing sequence **(C–K)**. **(A)** von Frey test/mechanical paw withdrawal thresholds. **(B)** Plantar heat test/radiant-heat induced paw-withdrawal latency. **(C–F)** Automated classification of voluntary behaviours in the spectroscopy apparatus. The parameters are ordered in general behaviours **(C)**, tracklength **(D)**, overall activity **(E)**, and velocity **(F)**. **(G)** 3 chamber apparatus/time spent in a compartment with an unknown animal. **(H)** Elevated plus maze/time spent in open arm exploration. **(I)** Splash-test/time in induced grooming behaviour. **(J)** Saccharine preference. **(K)** Nest building scoring (median ± 95% confidence interval, Kruskal-Wallis test with Dunn's multiple comparison test). Data are expressed as mean ± SEM and analysed using a RM two-way ANOVA **(A,B)**, followed by Bonferroni's correction (time and treatment as dependent variables). For single time point behavioural tests, a one-way ANOVA with Bonferroni's correction was used (**C–K**, *n* = 8 for vehicle control, *n* = 8 for the nitroglycerine cephalgia model, *n* = 8 for the nitroglycerine/sumatriptan group). * indicates a significant difference to the vehicle control (*p* < 0.05). For detailed *p*-values of group comparisons, see Table 1.

We then tested a model of nitroglycerine-induced acute cephalgia (22) in the behavioural testing sequence (Figure 1A). Rats received single *i.p*. injections of a nitroglycerine-solution. After 1 h, a subgroup of animals received a single administration of the vasoconstrictor sumatriptan as a pharmacological treatment. Nitroglycerine induced a significant decrease both in mechanical paw withdrawal thresholds (*p* < 0.0001) and thermal withdrawal latencies (*p* = 0.0098) 2 h post injection (Figures 4A,B; Table 1). The administration of sumatriptan significantly reduced these effects (*p* = 0.082 and *p* > 0.99; Figures 4A,B; Table 1). Rats which received nitroglycerine injections showed a significant decrease in open arm exploration on the elevated plus maze (*p* = 0.0036) which was not improved by sumatripan (*p* = 0.014; Figure 4H; Table 1). Other parameters were not altered by the two treatment regimens (*p* > 0.05; Figure 4; Table 1).

### Impact of Gabapentin and Aprepitant on CCI-Induced Behavioural Responses

**Figure 5:**
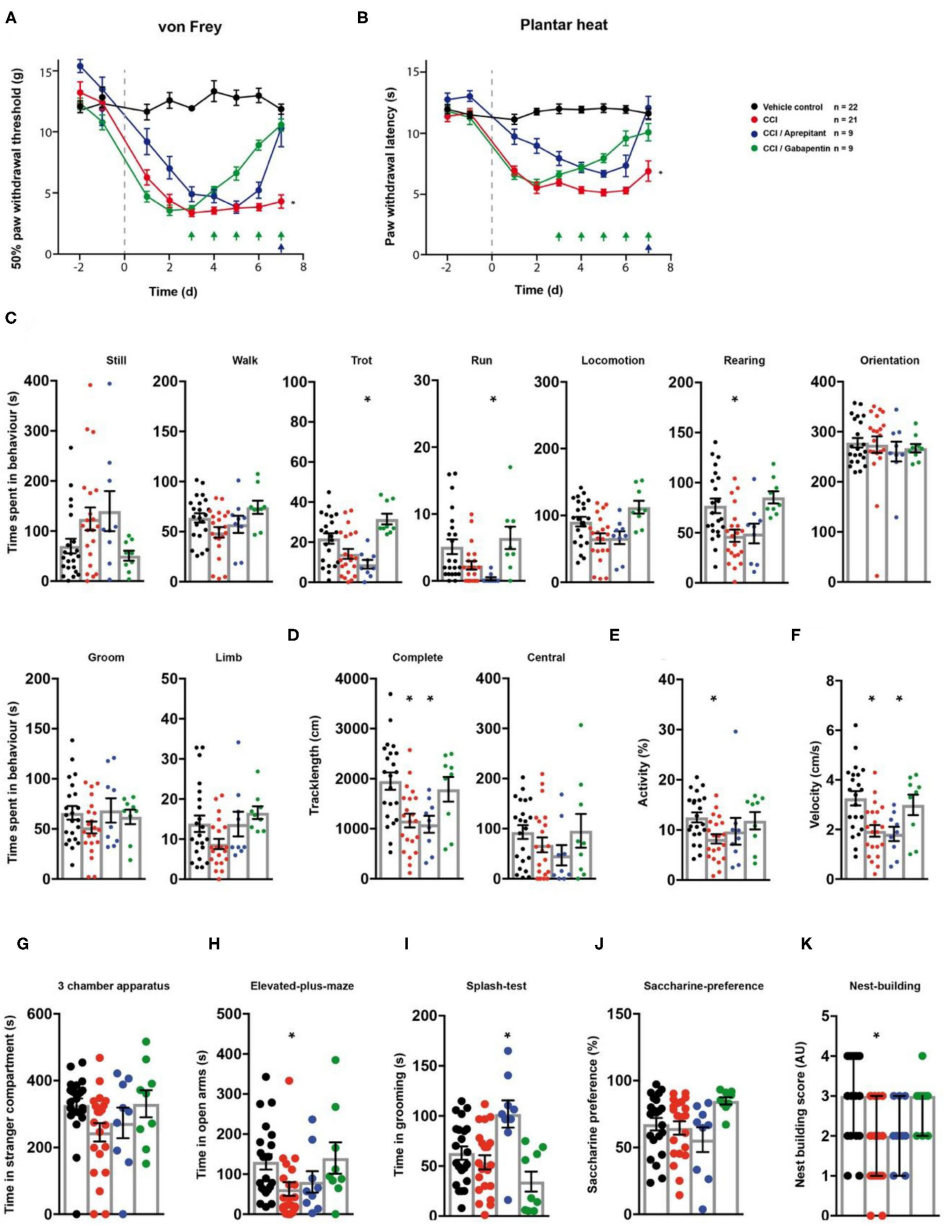
Gabapentin and aprepitant have distinct effects on CCI-induced neuropathic symptoms. CCI- (red traces/columns), vehicle- (black trace/columns), CCI/gabapentin- and CCI/aprepitant treated (blue traces/columns) rats were tested on two baseline- and 7 post surgery time points for their responses to mechanical- and heat stimuli. CCI surgery was performed at time point 0, as indicated by the grey dashed line **(A,B)**. Injection time points of gabapentin (green) and aprepitant (blue) are indicated by arrows on the x-axis **(A,B)**. At the last testing day post CCI surgery, all groups were subjected to the testing sequence **(C–L)**. **(A)** von Frey test/mechanical paw withdrawal thresholds. **(B)** Plantar heat test/radiant-heat induced paw-withdrawal latency. **(C–F)** Automated classification of voluntary behaviours in the spectroscopy apparatus. The parameters are ordered in general behaviours **(C)**, tracklength **(D)**, overall activity **(E)**, and velocity **(F)**. **(G)** 3 chamber apparatus/time spent in a compartment with an unknown animal. **(H)** Elevated plus maze/time spent in open arm exploration. **(I)** Splash-test/time in induced grooming behaviour. **(J)** Saccharine preference. **(K)** Nest building scoring (median ± 95% confidence interval, Kruskal-Wallis test with Dunn's multiple comparison test). Data are expressed as mean ± SEM and analysed using a RM two-way ANOVA **(A,B)**, followed by Bonferroni's correction (time and treatment as dependent variables). For single time point behavioural tests, a one-way ANOVA with Dunnett's correction was used [**C–K**, *n* = 22 for the control-group, *n* = 21 for the CCI model, *n* = 9 for the CCI/aprepitant and the CCI/gabapentin. * indicates a significant difference to the control-group (*p* < 0.05)]. For detailed *p*-values of group comparisons, see Table 1.

Next, we assessed the effect of gabapentin and aprepitant on the behaviour of rats in a model of peripheral neuropathy. We surgically induced a unilateral chronic constriction injury (CCI) of the sciatic nerve (28). A cohort of animals with CCI received *i.p*. injections of gabapentin over a 5 day period. Another group of CCI-rats received a single aprepitant *i.p* injection at the last day of testing (Figure 1A). Rats which underwent CCI surgery showed significantly reduced mechanical paw withdrawal thresholds and significantly reduced thermal withdrawal thresholds 7 days post-surgery (both *p* < 0.0001; Figures 5A,B; Table 1). Animals receiving either gabapentin or aprepitant did not differ in the threshold tests from the control group (*p* = 0.91 and *p* = 0.55; Figures 5A,B; Table 1). Furthermore, rats which underwent CCI surgery, showed significantly altered behaviour in the spectroscopy apparatus 7 days after nerve ligation (Figures 5C–F; Table 1). A significant decrease in time spent rearing (*p* = 0.001; Figure 5C; Table 1) and an altered locomotive behaviour was observed: this includes a significant reduction of the time spent in locomotion (*p* = 0.04), of the general track length in the complete field (*p* = 0.001), overall activity (*p* = 0.025), and velocity (*p* = 0.001; Figures 5C–F, Table 1). Gabapentin mitigated the CCI-induced effects on rearing and all locomotion parameters (*p* > 0.05). Aprepitant improved general locomotion and overall activity (*p* > 0.05), but failed to counteract the CCI induced effects on track length and velocity parameters (*p* > 0.05; Figures 5C–F; Table 1). In the tests for affective behaviour, rats subjected to CCI depicted a significant reduction of open arm exploration on the elevated plus maze, 7 days after surgery (*p* = 0.04; Figure 5H; Table 1). This effect was reduced by gabapentin (*p* = 0.98) or aprepitant administration (*p* = 0.36; Figure 5H, Table 1). In addition, CCI significantly impaired the nest quality (*p* = 0.001), which was improved by gabapentin (*p* = 0.99) or aprepitant (*p* = 0.051; Figure 5K; Table 1). CCI alone or in combination with both pharmacological treatment regimens had no effect on the time spent in stranger compartments or on the saccharine preference (*p* > 0.05; Figures 5G,J; Table 1). Aprepitant administration induced a significant increase in grooming behaviour in the splash test after sucrose application, when compared to the control (Figure 5I, *p* = 0.012).

### Phenytoin Effects on Behaviour in Oxaliplatin-Induced Neuropathy

**Figure 6:**
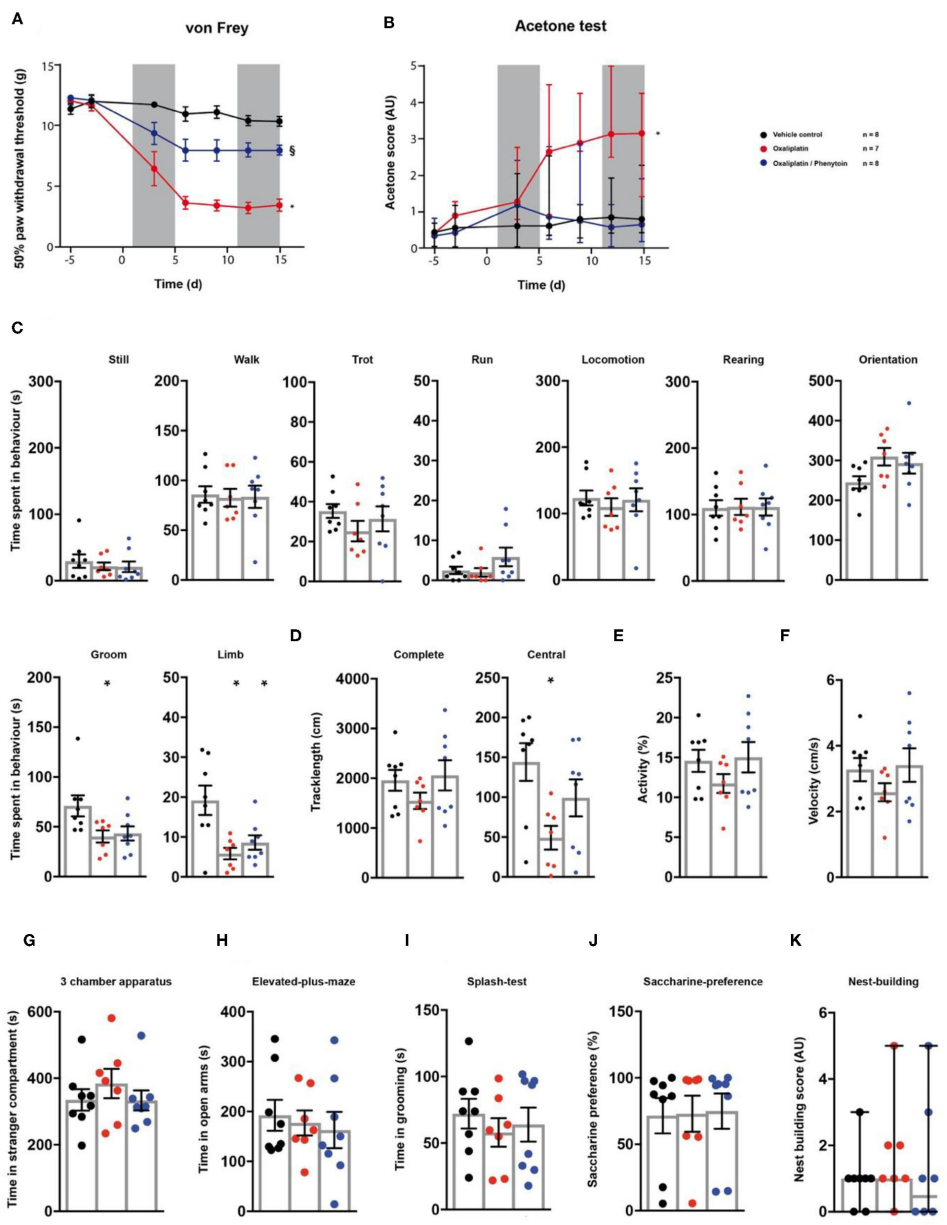
Effects of phenytoin on oxaliplatin induced symptoms. Oxaliplatin- (red trace), vehicle- (black trace), and oxaliplatin/phenytoin-treated (blue trace) rats were tested on two baseline- and 5 time points post injection for their responses to mechanical- and cold stimuli. Oxaliplatin injections were performed at time points 1–5 and 11–15, as indicated by the two grey fields **(A,B)**. Phenytoin was administered daily from time-point 1–15. At the last testing time point, all groups were subjected to the testing sequence **(C–L)**. **(A)** von Frey test/mechanical paw withdrawal thresholds. **(B)** Acetone-test induced responses (median and 95% confidence interval, non-parametric longitudinal analysis). **(C–F)** Automated classification of voluntary behaviours in the spectroscopy apparatus. The parameters are ordered in general behaviours **(C)**, tracklength **(D)**, overall activity **(E)**, and velocity **(F)**. **(G)** 3 chamber apparatus/time spent in a compartment with an unknown animal. **(H)** Elevated plus maze/time spent in open arm exploration. **(I)** Splash-test/time in induced grooming behaviour. **(J)** Saccharine preference. **(K)** Nest building scoring (median ± 95% confidence interval, Kruskal-Wallis test with Dunn's multiple comparison test). Data are expressed as mean ± SEM and analysed using a RM two-way ANOVA **(A)**, followed by Bonferroni's correction. For single time point behavioural tests, a one-way ANOVA with Bonferroni's correction was used (**C–K**, *n* = 8 for vehicle control, *n* = 7 for the oxaliplatin group, *n* = 8 for the oxaliplatin/phenytoin group). * indicates a significant difference to the vehicle control (*p* < 0.05); § indicates a significant difference to the oxaliplatin-group (*p* < 0.05). For detailed *p*-values of group comparisons, see Table 1.

Finally, we tested the model of oxaliplatin-induced chronic neuropathy in the behavioural array (23). Rats received cyclic oxaliplatin injections, closely mimicking clinical use of the chemotherapeutic oxaliplatin for colorectal-cancer treatment (38). A cohort of the animals subjected to oxaliplatin injections received an adjuvant phenytoin therapy, consisting of 15 daily *i.p*. injections (Figure 1A). Oxaliplatin treatment induced mechanical hypersensitivity (*p* < 0.0001; Figure 6A; Table 1), which was reduced by phenytoin administration (*p* = 0.0001; Figure 6A; Table 1). Further, oxaliplatin induced cold hypersensitivity, indicated by an increase in the acetone response score over the injection cycle (day 15: *p* = 0.006; Figure 6B; Table 1). Adjuvant phenytoin co-application fully prevented cold hypersensitivity (*p* = 1.0; Figure 6B; Table 1). In the spectroscopy task for the quantification of behavioural parameters, oxaliplatin-treated animals displayed a reduction in grooming (*p* = 0.036), limb-directed behaviour (*p* = 0.003) and track length in the central field (*p* = 0.011; Figures 6C,D; Table 1). Phenytoin treatment improved grooming behaviour (*p* = 0.052) and the track length (*p* = 0.292), but failed to counteract the oxaliplatin-induced decrease in limb-directed behaviour (*p* = 0.015, Figures 6C,D; Table 1). Oxaliplatin and oxaliplatin/phenytoin treatment did not alter the behaviour of the animals in the other tests (*p* > 0.05; Figures 6G–K; Table 1). Together, these results show a potent analgesic impact of phenytoin on oxaliplatin-induced mechanical- and cold hypersensitivity, and a modest effect on altered voluntary/spontaneous behaviour.

### Behavioural Parameter Evaluation

**Figure 7:**
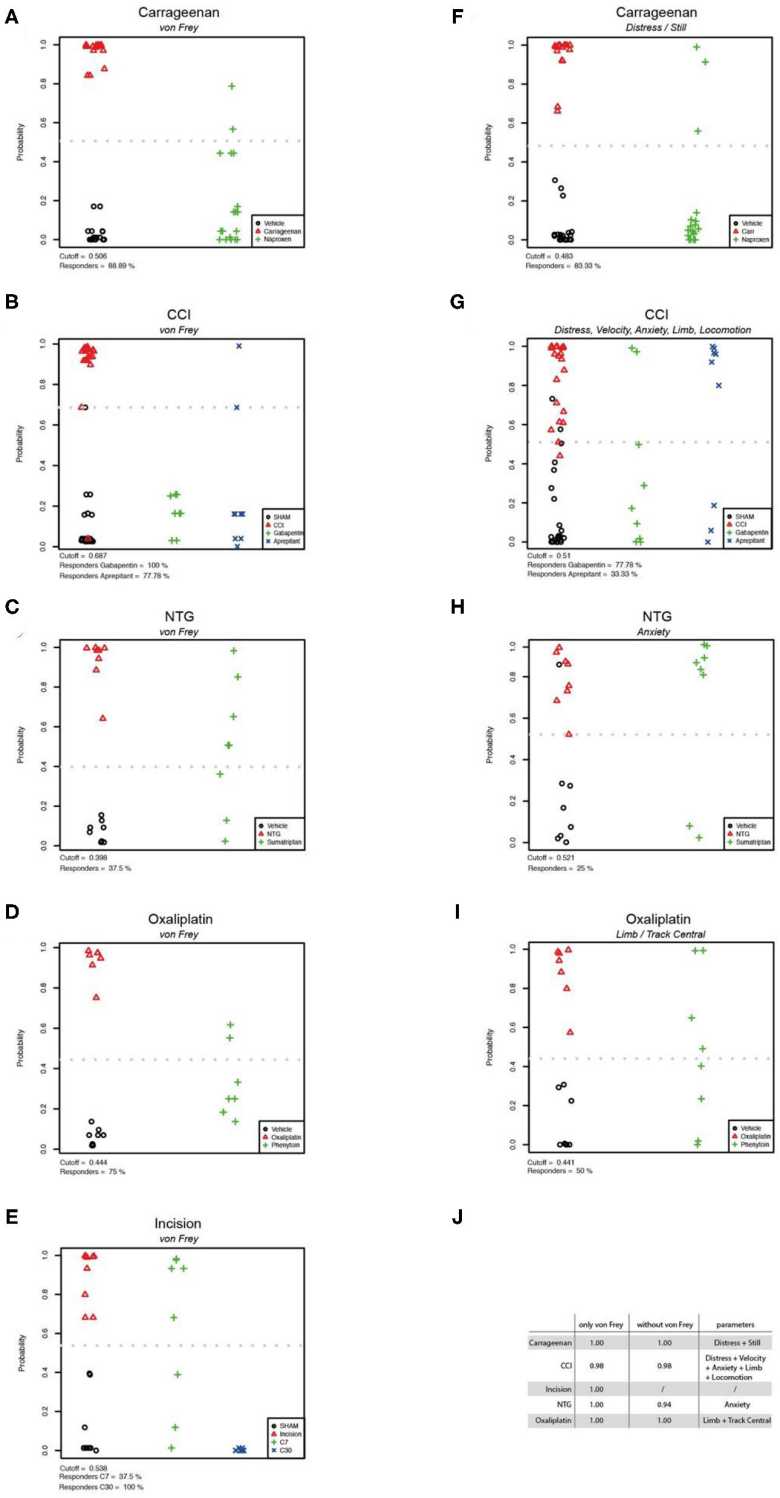
Statistical modelling of group separation by von Frey testing and non-threshold behavioural tests. Firth logistic regression models and the analysis (DFA) of pain models and their corresponding control group (*n*: vehicle = 67, Carrageenan = 18, CCI = 22, Oxaliplatin = 7, Cephalgia = 8, Incision = 9). The hollow red triangles represent the predictions in the pain group. The green pluses (and blue crosses) represent the predictions for the treatment group(-s) based on the Firth regression model. The cutoff is displayed as a grey dotted line **(A–I)**. The results of Firth logistic regressions with von Frey can be seen in figures **(A–E)**. The results of Firth regression without von Frey are presented in figures **(F–I)**. The variables have been chosen using forward selection and are listed in table **(J)**. Here the Area Under the Curve (AUC) values are illustrated, which are a measure of the accuracy of classifiers obtained by Firth logistic regression.

To classify the behavioural parameters used in this study, we performed a firth regression analysis which allowed the evaluation of discrimination efficacy between the vehicle control groups and the pain models based on different behavioural tests. First, we evaluated von Frey as parameter for group separation between control- and pain model-cohorts, and calculated the responder rate of rats which could be classified to the control-group after analgesic treatment (Figures 7A–E). The von Frey test showed a high accuracy of separation in the carrageenan, incision, oxaliplatin, and nitroglycerine-induced cephalgia models (AUC = 1.00; Figure 7J). In the CCI model an AUC of 0.98 was detected with von Frey testing (Figure 7J). We used forward selection to determine affective/voluntary variables with similar accuracy to von Frey testing in the different pain models (Figure 7J). In the carrageenan model for inflammatory pain, the combination of distress and still showed an AUC of 1.00 (Figures 7F,J). In the CCI model for neuropathic pain, the combined parameters distress, velocity, anxiety, limb, and locomotion depicted an AUC of 0.98 (Figures 7G,J). In the nitroglicerine-induced cephalgia model, the elevated plus maze was detected as the parameter with the highest accuracy (AUC = 0.94; Figures 7H,J). In the oxaliplatin model for chemotherapy induced neuropathy, a combination of parameters (limb and track central) illustrated the highest accuracy (AUC = 1.00; Figures 7I,J).

Together, these data illustrate that von Frey is a consistent parameter in terms of accuracy to discriminate between control- and pain-groups across different pain models. In addition, our data show that this accuracy can be met by combining voluntary/affective parameters of clinically-related behavioural assays.

## Discussion

Most of the presently used animal models of acute and chronic pain were developed on the basis of enhanced sensitivity to sensory stimuli, such as measured in the reflexive von Frey-test. It is thus not surprising that these pain models all share mechanical and/or thermal hypersensitivity. Consistently, the von Frey test revealed mechanical hypersensitivity in all pain-groups when compared to the control group in the present study. To broaden the assessment of pathological pain states in rodents and the effect of analgesics, we employed a battery of tests for each pain model and treatment.

### Behavioural Considerations

The most common symptom studied and reported in preclinical rodent pain models is the enhanced responsiveness to mechanical- and/or heat stimuli. This also reflects the historical co-evolution of nocifensive withdrawal tests and animal models of chronic pain (39, 40), where the behavioural pathology was primarily validated by tests for sensory gain (28, 41–44). The most prevalent and disturbing symptom in the majority of chronic pain patients is, in contrast, ongoing pain (45, 46). To study ongoing pain in rodents, we used an automated classification of rodent behaviours (30). Some, but not all preclinical reports have observed altered parameters of spontaneous/voluntary behaviour in rodents, under pathological pain conditions (47, 48). It is likely that the absence of standardised testing protocols, observation parameters, and assessment procedures contribute to conflicting observations in the literature (49). Evidence from lesion studies of the anterior cingulate cortex, a structure implied in the processing of affective pain components and ongoing pain, suggests that altered paw-directed behaviour, weight bearing, and locomotion-parameters are useful markers for ongoing pain (50–52). Besides ongoing pain, manifestations of anxiety, depression, and distress are common comorbidities in human pain patients (12, 53, 54). In order to also assess these comorbidities, we selected behavioural tests with a proven face validity which are well-anchored in the basic research (31, 33, 55–57). We composed the sequence of individual tests according to the expected stress-burden on the animals, starting with the least strenuous task. Although we cannot exclude the possibility of earlier tests affecting the behaviour of subsequent tests, we expect these effects to be equal across all models and treatment groups, since the testing regime was always performed in the same sequence. Moreover, earlier studies have shown that sequenced testing has no effect on relevant parameters of the elevated plus maze, open field test, and other cognitive behavioural tasks (58, 59). Of note, we did not observe any significant differences between rats tested on a one-test-per-day basis and the general vehicle population in which all tests were performed on the same day.

### Symptom Manifestations and Analgesic Effect in the Pain Models

Distinct chronic pain pathologies may share similar molecular- and cellular mechanisms, but can vary in their pattern of clinical manifestation (40, 46, 60–63). In the clinics, this patterned development of symptoms allows the detection of patient clusters even within the same pain aetiology (64). Accordingly, we found that single parameters of rat behaviour were modulated in a pain aetiology-dependent manner. For example, CCI-induced neuropathy and carrageenan-induced inflammation considerably altered locomotor parameters of the animals, which is in agreement of previous literature (30, 65–68). In contrast, oxaliplatin-induced neuropathy reduced grooming and limb-directed behaviours, but not any off the locomotor related parameters. Voluntary and spontaneous behaviours have not previously been studied extensively in rodent models of oxaliplatin-induced neuropathy. But, in line with our data, paclitaxel, a chemotherapeutic which also induces sensory neuropathy in rodents, has been shown to decrease voluntary burrowing behaviours (69). In the incision model of acute postsurgical pain altered weight bearing has been noted up to 2 days after hind-paw incision (21). We did, however, not observe any significant changes in voluntary and spontaneous behaviour after incision surgery, suggesting that the measured parameters are not altered by altered weight bearing. Similarly to our own observations, it has been reported that hind-paw incisions have no effect on motor activity in rats (70). It is also likely that the testing time-point 48 h post incision is just outside the time-course of on-evoked guarding pain behaviour in this model (71). Nitroglycerine-induced-cephalgia in rats has been shown to induce decreased locomotion and an increased anxiety-like behaviour (72, 73). In the testing sequence, we observed no changes in locomotor behaviour, but nitroglycerine administration increased avoidance of the open arms of the elevated plus maze. In general, we also observed a pain model-dependent development of affective behavioural parameters. In contrast, we did not observe any model-induced changes in the relevant parameters for the splash test or saccharine preference.

We chose Naproxen, codeine, sumatriptan, and gabapentin for aetiology specific analgesic treatment based on the low clinical NNT values. Our data show that all compounds had an overall beneficial effect on the behavioural parameters in the corresponding pain models. Naproxen, an unselective cyclooxygenase inhibitor, shows a high efficacy in the treatment of inflammatory pain-related pathologies in humans (74). This is also reflected in rat models of inflammation, where naproxen treatment reduced hypersensitivities, normalises gait parameters and other non-evoked behavioural parameters in the present and other studies (30, 75, 76). In our study, naproxen normalised hypersensitivity, locomotion parameters, and distress symptoms in the carrageenan induced inflammatory pain model. These effects resulted in an 89% responder rate measured *via* von Frey and 83% responders assessed *via* distress and still behaviour. Sumatriptan, an antimigraine compound, showed a low 37% responder rate measured *via* von Frey in the nitroglycerin induced cephalgia model, which does not match its high efficacy in treating cephalgia in rodent research models (22, 75). Compared to placebo, the opiate codeine alleviates mild post-surgical pain in clinical settings in some patients (77). In rodents, codeine is shown to reduce mechanical hypersensitivities in a dose dependent manner (78). Here, the lower codeine dose (7 mg/kg), the equivalent dose of codeine used in the clinics, had no significant influence on the threshold tests, but resulted in an overall 37.5% responder rate in the combined testing. The highest dose (30 mg/kg), in contrast, led to a significant reduced responsivity in the thresholds test and to a 100% responder rate. This high effect of codeine at 30 mg/kg, compared to 7 mg/kg, on the thresholds tests may, however, be related to side-effects on motor behaviour as the administration of codeine at 30 mg/kg also increased still behaviour which in turn can increase apparent mechanical thresholds. Gabapentin is recommended as first line treatment in humans for neuropathic pain and classified with a level A rating for treatment efficacy (79). Gabapentin had a significant therapeutic effect on all altered behavioural parameters in the present study, resulting in a responder rate of 100% assessed *via* von Frey and a somewhat a lower responder rate of 78% when assessed by voluntary-affective parameters. Similarly, gabapentin has been shown to significantly alleviate sensory gain, anxiety, and normalise tunnel burrowing behaviour in previous studies of neuropathic pain (18, 80–82). Earlier rodent studies strongly implied neurokinin-1-receptor antagonists as effective treatment of pathological pain but did not yield promising results in a clinical trial (4). Our results on aprepitant might reflect on this dynamic, with a high responder rate in the von Frey testing of 100% and a very low responder rate of 33% in the affective/voluntary parameters.

Based on previous preclinical data, phenytoin, an inhibitor of voltage-gated sodium channels, has been suggested as a potential pharmacological compound to treat oxaliplatin-induced neuropathic pain (25, 26, 83). We observed a phenytoin-mediated decrease of mechanical- and cold hypersensitivity (von Frey responder rate 75%), but a small effect on abnormal voluntary and spontaneous behaviour (voluntary behaviour responder rate 50%). This pattern of phenytoin treatment might reflect its inability to prevent ongoing pain-related spontaneous ectopic discharges in the soma of primary afferents. These discharges depend in part on voltage-gated potassium channels (K_V_'s), and are therefore unaffected by phenytoin (84–86). Accordingly, clinical phenytoin treatment of diabetic neuropathy, which also involves altered expression of K_V_'s, was shown to be inefficacious (87).

Together our results indicate that the von Frey test has the expected high accuracy for the differentiation between rat models of the included pathological pain and control groups. This likely reflects that mechanical hypersensitivity testing had historically been used as validation parameter during the development of the various animal models of pain. The distinct alterations in voluntary/affective parameters likely relate to differences in aetiologies and individual time-courses of the pain models studied. All analgesics administered to the respective pain models induced an increase in mechanical thresholds, but showed different effects on voluntary and affective behaviours. This observation is further expanded by the firth regression analysis, which suggests individual sets of affective/voluntary behaviours as parameters with the highest discriminatory accuracy. The responder rate, when assessed with these parameters only is consistently lower when compared to the responder rate determined *via* mechanical threshold testing. Thus, for identifying clinically successful analgesics, the attenuation of mechanical hypersensitivity in preclinical rodent testing is an useful readout (88), that should be complemented rather than substituted by additional voluntary/affective parameters.

### Limitations

The present study was on male rats. It should be noted, however, that increasing evidence points to different mechanisms of pain processing in male and female rodents which have been shown to also affect associated behaviours (89–91). In the present study behavioural assessment lasted for a maximum of 15 days, and thus could not capture any potential behavioural changes, such as depressive- or anhedonic states, that might have developed at later time points (92). Furthermore, we could not include all possible tests that might prove useful in evaluating pain-related behaviour in rodents. For example, burrowing, a voluntary behaviour observed in rodents has emerged as a non-stimulus evoked test for “daily-living activity” and a surrogate for pre-clinical pain assessment (56, 82, 93). Similarly, the grimace scale which is increasingly utilised in rodents and other species to assess ongoing pain, was not utilised in this study (88, 94). The interpretation of animal behaviour seen in the spectroscopy analysis likely reflects strong exploratory behaviour of rats in a foreign environment without cage-mates could be classified as voluntary-rodent specific behaviour. Home cage activity analysis would allow longer observation periods with cage-mates in a continuous light/dark cycle (95, 96).

### Preclinical- and Clinical Implications

Mechanical threshold testing is the default testing method to assess pathological rodent pain states and is historically linked to the pain models which are used in today's pre-clinical research. Collectively, our data further support the notion that von Frey mechanical threshold testing is a useful parameter to assess pathological pain-states of different aetiologies in rats. This read-out is, however, intrinsically one-dimensional suggesting that mechanical threshold tests should be supplemented by pathology-specific test of voluntary and adversive pain behaviour to best match the clinical situation of chronic pain patients.

## Data Availability Statement

The raw data supporting the conclusions of this article will be made available by the authors, without undue reservation.

## Ethics Statement

The animal study was reviewed and approved by Austrian Ministry for Science and Research (BMWF).

## Author Contributions

PD, AM, and JS designed research and wrote the manuscript, with input from the other authors. PD, AM, KG, and AB conducted experiments and analysed data. DR performed firth regression analysis. All authors discussed the results and approved the final version of the manuscript.

## Conflict of Interest

Since completion of the experimental work, PD is employed at MSD. The remaining authors declare that the research was conducted in the absence of any commercial or financial relationships that could be construed as a potential conflict of interest.

## Supplementary Material

Supplementary Figure 1 Validation of the behavioural classification of the spectroscopy apparatus. A total of 8 naïve rats were tested for 10 min each in the spectroscopy apparatus. The videotaped behaviour of the animals was manually classified by an experimenter and compared to the classification performed by the categorisation software. **(A)** Bar graph illustrates the quantified behaviours by an experimenter (blue) and by the algorithm of the spectroscopy apparatus (red). No significant difference of the parameters was detected (*p* > 0.05 for all comparisons). Data are expressed as mean ± SEM, and were analysed by a one-way ANOVA with Bonferroni's correction. **(B)** Correlation of experimenter- and computer-quantification of mean parameter values observed in the spectroscopy apparatus (r = 0.99; *R*^2^ = 0.98, *p* < 0.01). **(C)** Correlation of experimenter and computer quantification of individual parameter values observed in the spectroscopy apparatus (r = 0.97, *R*^2^ = 0.94, *p* = 0.01). **(A,B)** Data were analysed using a correlation-analysis to determine the Pearson-coefficient (r) and the coefficient of determination (*R*^2^). **(D)** Example plot of the detected behaviour of a single animal over a 10 min period (x-axis) in the spectroscopy apparatus. Red rectangles indicate the observed parameter, its time point and extent over time as classified by the spectroscopy algorithm. Blue squares indicate the observation of the experimenter via manual categorization.
