# Supplementary figures and images for: Spontaneous, Voluntary, and Affective Behaviours in Rat Models of Pathological Pain
Source: Front Pain Res (Lausanne). 2021 Jul 1;2:672711. doi: 10.3389/fpain.2021.672711 (PMC8915731; doi:10.3389/fpain.2021.672711)

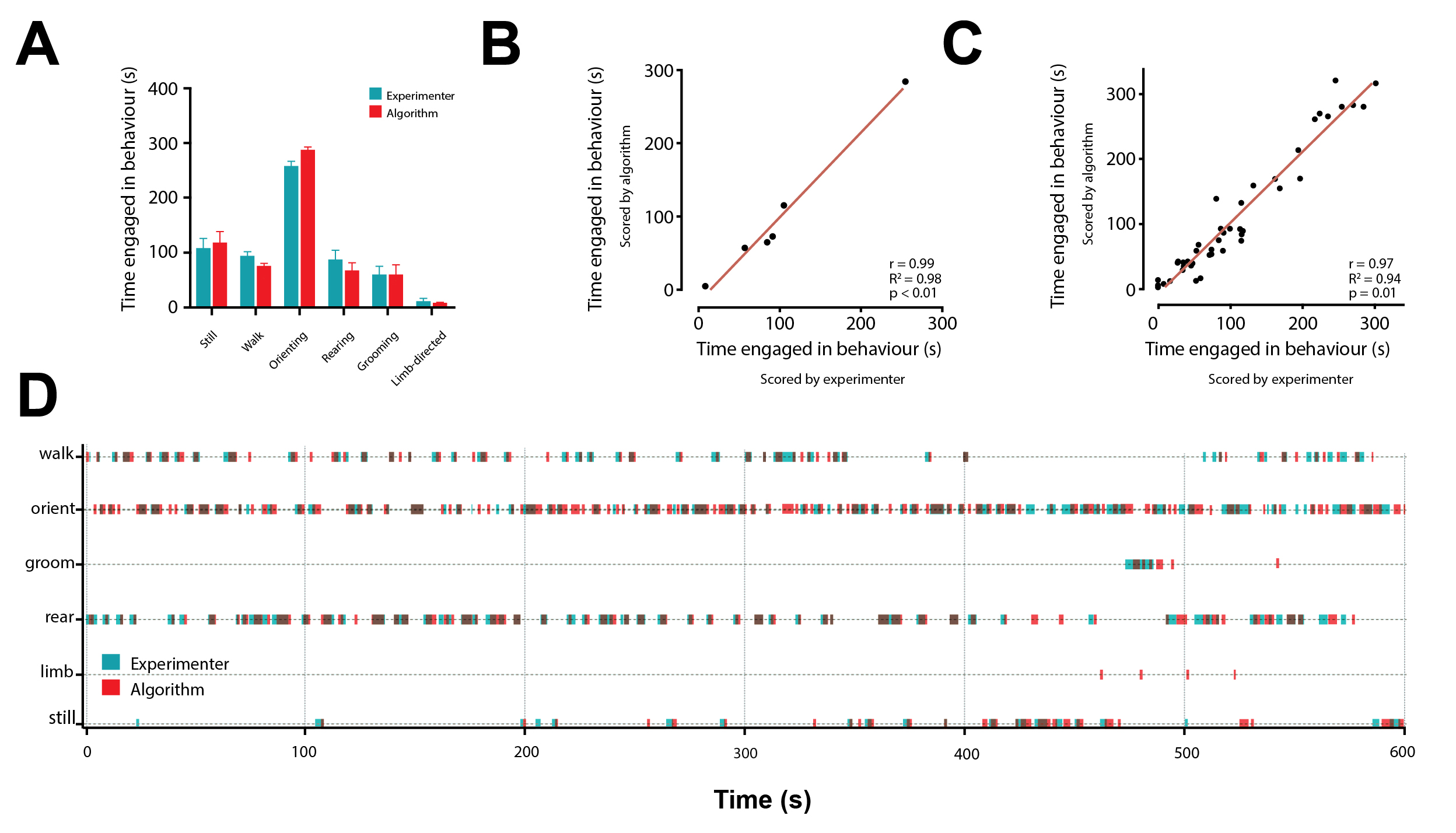

Supplement: Supplementary Figure 1 — Validation of the behavioural classification of the spectroscopy apparatus. A total of 8 naïve rats were tested for 10 min each in the spectroscopy apparatus. The videotaped behaviour of the animals was manually classified by an experimenter and compared to the classification performed by the categorisation software. (A) Bar graph illustrates the quantified behaviours by an experimenter (blue) and by the algorithm of the spectroscopy apparatus (red). No significant difference of the parameters was detected (p > 0.05 for all comparisons). Data are expressed as mean ± SEM, and were analysed by a one-way ANOVA with Bonferroni's correction. (B) Correlation of experimenter- and computer-quantification of mean parameter values observed in the spectroscopy apparatus (r = 0.99; R2 = 0.98, p < 0.01). (C) Correlation of experimenter and computer quantification of individual parameter values observed in the spectroscopy apparatus (r = 0.97, R2 = 0.94, p = 0.01). (A,B) Data were analysed using a correlation-analysis to determine the Pearson-coefficient (r) and the coefficient of determination (R2). (D) Example plot of the detected behaviour of a single animal over a 10 min period (x-axis) in the spectroscopy apparatus. Red rectangles indicate the observed parameter, its time point and extent over time as classified by the spectroscopy algorithm. Blue squares indicate the observation of the experimenter via manual categorization. [file Image_1.JPEG]
